# Stercoral Colitis: An Unexpected Presentation in a Young Adolescent

**DOI:** 10.7759/cureus.55835

**Published:** 2024-03-09

**Authors:** Max J Jones, Esther Adeyemi, Sarkis Kouyoumjian

**Affiliations:** 1 Emergency Medicine, Wayne State University Detroit Medical Center, Detroit, USA

**Keywords:** treatment strategies, diagnostic challenges, pediatric patients, fecaloma, stercoral colitis

## Abstract

Stercoral colitis is an uncommon inflammatory condition primarily affecting individuals with chronic constipation, immobilization, and advanced age, occasionally manifesting in pediatric patients. It arises from the accumulation of impacted fecal matter, leading to colonic distension and subsequent fecaloma formation, culminating in pressure necrosis and perforation. Mortality rates can exceed 60% in vulnerable populations due to complications such as colonic perforation and ischemia.

Presented is the case of a 14-year-old female with stercoral colitis, initially presenting with diarrhea, abdominal pain, and metabolic acidosis. Despite improvement followed by a sudden deterioration, diagnostic challenges persisted, highlighting the complexity of diagnosing this condition, especially in pediatric cases. Key diagnostic criteria include vague abdominal symptoms, leukocytosis, and elevated inflammatory markers, alongside potential metabolic derangements. Imaging modalities, such as abdominal CT scans, aid in diagnosis, delineating features like colonic distension and wall thickening.

Treatment strategies encompass aggressive bowel disimpaction, with endoscopic or surgical interventions reserved for refractory cases or perforations. Recognition of stercoral colitis is crucial for timely intervention, given its significant morbidity and mortality. Although typically associated with elderly or bedbound patients, the condition can also affect younger individuals, emphasizing the importance of considering it in the differential diagnosis, particularly in cases of chronic constipation. Integration of diagnostic imaging techniques facilitates accurate diagnosis, guiding appropriate therapeutic interventions and potentially mitigating adverse outcomes.

## Introduction

Stercoral colitis is a rare inflammatory form of colitis, mainly impacting populations who are plagued by chronic constipation, bedbound or nursing home residents, elderly individuals, and occasionally pediatric patients. This specific form of colitis occurs when impacted fecal material leads to the distention of the colon and subsequent fecaloma formation, which encourages pressure necrosis and perforation. Moreover, colonic distention can lead to vascular compromise and ischemic colitis [1].

While multiple areas of the colon can become ulcerated due to fecaloma formation, the most common area of ulceration is the sigmoid colon and rectum. Due to the complications that can arise, caused by colonic perforation and ischemia, the mortality rate can be upwards of 60% in vulnerable patient populations [2].

## Case presentation

A 14-year-old female was transferred to a large, pediatric tertiary care hospital from an outlying hospital. Upon arrival, the patient complained of diarrhea with diffuse abdominal pain. Additionally, labs from the transferring facility suggested metabolic acidosis.

A history obtained in the Emergency Department revealed that the patient's illness began three days prior with nausea and vomiting, progressing to diarrhea and diffuse abdominal pain on presentation. She initially improved, demonstrated by an overall decrease in her nausea, abdominal pain, and diarrhea. However, on the day of admission, the patient had a marked decline in her overall condition and appearance, having non-bilious, non-bloody vomiting and loose stools every one to two hours. Per her mother, she later became pale, limp, and unresponsive to stimuli, which prompted the family to bring her to the outside facility. Prior to this decline, the mother reports the patient had a normal appetite, but was having problems with intermittent constipation.

Reviewing the patient’s past medical and surgical history, she was found to have been diagnosed with Blount’s disease, a disease affecting the growth plates around the knee, causing the medial portion of the growth plate to stop while the lateral portion continues to grow, causing a bowing of the knee. Additionally, she was found to have had previous episodes of constipation requiring surgical evacuations. She had an unremarkable social history, denied taking any medications, and had no recent travel. Moreover, the mother mentioned the patient may have diverticulitis; however, this remained unconfirmed. A review of systems revealed generalized abdominal pain and loose stools for the past two days, with nothing else significant of note. On exam, the patient was hypotensive with a blood pressure of 84/47 mmHg, tachycardic at 164 bpm, tachypneic at 30 bpm, afebrile at 36.9 C, and 97% oxygen saturation on room air. Treatment at the transferring facility involved three boluses of intravenous fluids as indicated by the hypotension. A fourth bolus was started upon arrival at the hospital with no improvement in blood pressure. Subsequently, epinephrine was then added to augment blood pressure.

**Figure 1:**
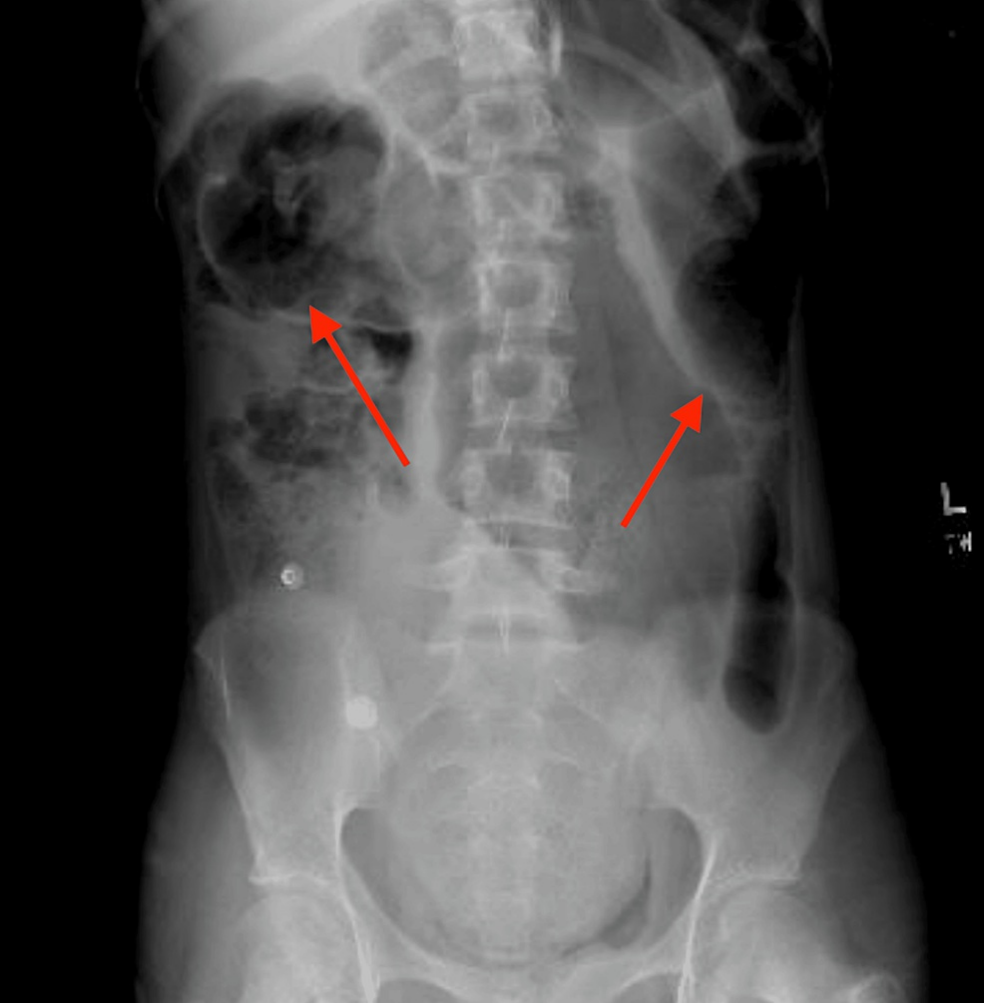
Radiograph indicating dilated loops of bowel consistent with a possible obstruction.

**Figure 2:**
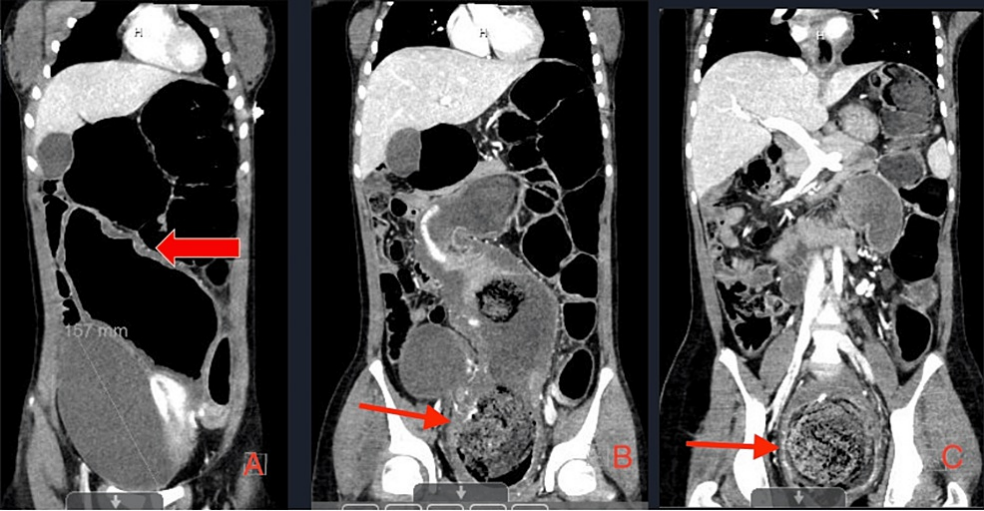
CT of the abdomen and pelvis showing diffusely dilated loops of bowel with evidence of a fecaloma. Image A demonstrates areas of dilated loops of the bowel with the red arrow pointing to a thickened section of the intestinal wall, which is suggestive of stercoral colitis. Image B demonstrates dilated loops of the bowel with the red arrow pointing to a large, obstructing fecaloma. Image C further demonstrates dilated loops of the bowel with the red arrow pointing to the obstructing fecaloma in greater detail.

A physical exam revealed a constitutionally ill-appearing patient who was crying in pain. A neurologic exam revealed no focal deficits with no meningeal signs and normal mentation. The patient was normocephalic with no trauma, the tympanic membranes were clear bilaterally with no swelling or redness. The pupils were equal in size and reactive to light. The nose had no nasal flaring or rhinorrhea, with moist mucus membranes. The neck was supple and non-tender, with no lymphadenopathy. There was presenting tachycardia with no murmurs, rubs, or gallops, with brisk capillary refill. Lungs were clear to auscultation with no wheezing, retractions, grunting, or stridor. The skin was dry with no erythema or rashes. The abdomen was soft and distended, with generalized tenderness on palpation, no guarding or rebound tenderness, and no hepatosplenomegaly appreciated. Laboratory findings, noted in Table 1, were significant for an elevated creatine kinase (CK), C-reactive protein (CRP), and troponin, due to a possible ischemic episode in the colon. Blood cultures revealed no growth after five days. Imaging is shown in Figures 1-2, and the patient was subsequently diagnosed with stercoral colitis.

**Table 1:** Laboratory results MCV: Mean corpuscular volume, MCH: Mean corpuscular hemoglobin, MCHC: Mean corpuscular hemoglobin concentration, RDW: Red cell distribution width, ALT: Alanine aminotransferase, AST: Aspartate aminotransferase, CK: Creatine kinase

Parameters	Patient Values	Reference Range
WBC	10,200 mm^3^	4,500-11,000 mm^3^
RBC	4.72 million/mm^3^	3.5-5.5 million/mm^3^
Hemoglobin	12.5 g/dL	12.0-16.0 g/dL
MCV	75.6 µm^3^	80-100 µm^3^
MCH	26.5 pg/cell	25-35 pg/cell
MCHC	35% Hb/cell	31-36% Hb/cell
RDW	13.90%	<14%
Platelets	184,000 mm^3^	150,000-400,000 mm^3^
ALT	48 U/L	10-40 U/L
AST	148 U/L	12-38 U/L
Alkaline Phos	120 U/L	25-100 U/L
CK	8894 U/L	30-145 U/L
Total Protein	49 g/dL	6.0-7.8 g/dL
Albumin	2.7 g/dL	3.5-5.5 g/dL
Troponin	161 ng/dL	<0.04 ng/dL
C-Reactive Protein	327 mg/dL	<1 mg/dL
Pregnancy (Serum)	Negative	Negative
Lipase	10 U/L	10-40 U/L
Sodium	126 mEq/L	136-146 mEq/L
Potassium	3.9 mEq/L	3.5-5.0 mEq/L
Chloride	101 mEq/L	95-105 mEq/L
Carbon Dioxide	12 mEq/L	23-29 mEq/L
Anion Gap	13 mEq/L	4-12 mEq/L
Random, Non-Fasting Glucose	89 mg/dL	<140 mg/dL
Urea Nitrogen	61 mg/dL	7-18 mg/dL
Creatinine	0.97 ng/dL	0.6-1.2 mg/dL
Bilirubin - Total	0.97 mg/dL	0.1-1.0 mg/dL
Calcium	7.2 mg/dL	8.4-10.2 mg/dL
Magnesium	2.5 mEq/L	1.5-2.0 mEq/L
Phosphorus	3.8 mg/dL	2.8-4.5 mg/dL

Once admitted, the patient was continued on epinephrine and intravenous fluids. The patient was evaluated by surgical staff and an evacuation was completed in the operating room with no ischemic bowel or resection of the colon noted. While recovering, vasopressor support was discontinued due to the patient’s improved condition. After the surgical evacuation, the patient’s guardian decided to discharge her against medical advice. Oral amoxicillin was sent to the patient’s preferred pharmacy in order to ensure that she was able to complete the 10-day course of antibiotics. The patient’s family was also provided information about the local pediatric gastroenterology clinic. Due to the abrupt discharge of the patient and the acuity of her condition, a child protective case and a wellness check were initiated, with no other follow-up available.

## Discussion

A diagnosis of stercoral colitis in susceptible adult patients can be extremely difficult due to the multitude of nonspecific clinical symptoms, creating difficulty in differentiating between other similar presentations like inflammatory bowel disease or appendicitis. A majority of the presenting symptoms in stercoral colitis are vague complaints of abdominal pain, vomiting, nausea, distention anorexia, and constipation [3]. Images demonstrating distention of the affected colon segment greater than six centimeters with fecal material, wall thickening greater than three millimeters of the affected colon segment, and pericolonic fat tissue stranding can assist in a definitive diagnosis. Moreover, a pediatric patient’s difficulty in communicating the time course of the symptomology, and describing the precise symptoms that are occurring, among other barriers, and the overall rarity of stercoral colitis in pediatric populations makes for a difficult diagnosis.

Given the high morbidity of the disease, it is important to recognize the symptomology of stercoral colitis in order to initiate treatment and cease the progression of the disease in order to limit the necrosis and damage to vital tissue. Moreover, when examining patients suspected to have stercoral colitis, they typically present with vague physical symptoms, leukocytosis, and elevated inflammation markers like C-reactive protein. Additionally, they can present with increased serum lactic acid levels and metabolic acidosis depending on the level of necrosis present [4]. With the symptoms presented and lab results provided, the differential diagnosis can include diverticulitis, ulcerative colitis, bowel obstruction, bowel perforation, mesenteric ischemia, or malignancy.

Depending on the acuity of the patient’s presentation, it may be recommended to obtain an upright abdominal or a left lateral decubitus radiograph in order to identify any free air present, indicating a bowel perforation. Moreover, an abdominal CT scan with contrast may be helpful in assessing distention of the colon, wall thickening, or pericolonic fat tissue stranding [5-8].

When treating stercoral colitis, stable patients are able to be managed with an aggressive regimen of laxatives, enemas, and bowel disimpaction. However, if this treatment method is unsuccessful or the patient’s condition is a contraindication for the regimen, gastrointestinal (GI) endoscopic guided disimpaction or surgical operation may be indicated. Furthermore, if a perforation is present with systemic decompensation, then aggressive intravenous antibiotics and surgical resection are required [9,10].

## Conclusions

Stercoral colitis is a rare disease, however, the impact it can have on the overall health and mortality of a patient is quite significant. While this disease is typically seen in elderly, bed-bound patients, rare cases have been reported in pediatric patients, especially those with a history of chronic constipation. Additionally, with the symptoms and lab results presented that lead to a lengthy differential diagnosis, there is a high degree of difficulty when diagnosing stercoral colitis. Moreover, with the introduction of an abdominal CT scan being the gold standard for diagnosing the disease, it relieves some pressure on the diagnosing clinician when relying on physical exam and lab results alone. Furthermore, the treatment for stercoral colitis remains the aggressive laxative treatment for low acuity cases, while surgical disimpaction remains the current treatment for more severe cases, especially those presenting with fecalomas and necrosis.

## References

[1] Naseer M, Gandhi J, Chams N, Kulairi Z (2017). Stercoral colitis complicated with ischemic colitis: a double-edge sword. BMC Gastroenterol.

[2] Morano C, Sharman T (2023). Stercoral colitis. StatPearls.

[3] Thomas MJ, Nanagiri A, Duvidovich S, Levy L, Bamji N (2023). Mission disimpaction: endoscopic management of stercoral colitis in an adolescent. JPGN Rep.

[4] Ahmad H, Jannat H, Khan U, Ahmad N (2023). Stercoral colitis: a diagnostic challenge and therapeutic approach in an elderly patient with chronic constipation. Cureus.

[5] Tajmalzai A, Najah DM (2021). Stercoral colitis due to massive fecal impaction: a case report and literature review. Radiol Case Rep.

[6] Ünal E, Onur MR, Balcı S, Görmez A, Akpınar E, Böge M (2017). Stercoral colitis: diagnostic value of CT findings. Diagn Interv Radiol.

[7] Bae E, Tran J, Shah K (2024). Stercoral colitis in the emergency department: a review of the literature. Int J Emerg Med.

[8] Heffernan C, Pachter HL, Megibow AJ, Macari M (2005). Stercoral colitis leading to fatal peritonitis: CT findings. AJR Am J Roentgenol.

[9] Keim AA, Campbell RL, Mullan AF (2023). Stercoral colitis in the emergency department: a retrospective review of presentation, management, and outcomes. Ann Emerg Med.

[10] Canders CP, Shing R, Rouhani A (2015). Stercoral colitis in two young psychiatric patients presenting with abdominal pain. J Emerg Med.

